# Centrosomal protein 55 activates NF-κB signalling and promotes pancreatic cancer cells aggressiveness

**DOI:** 10.1038/s41598-017-06132-z

**Published:** 2017-07-19

**Authors:** Tao Peng, Wei Zhou, Feng Guo, He-shui Wu, Chun-you Wang, Li Wang, Zhi-yong Yang

**Affiliations:** 1 Department of Pancreatic Surgery, Union Hospital, Tongji Medical College, Huazhong University of Science and Technology, 1277 Jiefang Avenue, Wuhan, 430022 China; 2 Department of Emergency Surgery, Union Hospital, Tongji Medical College, Huazhong University of Science and Technology, 1277 Jiefang Avenue, Wuhan, 430022 China

## Abstract

Centrosomal protein 55 (CEP55) is a microtubule-bundling protein that participants in cell mitosis. It is overexpressed in several solid tumours and promotes the growth and invasion of cancer cells. However, the role of CEP55 in pancreatic cancer (PANC) remains unclear. Herein, upregulated expression of CEP55 (associated with poor prognosis) was detected in PANC using quantitative real-time reverse transcription PCR, western blotting, and immunohistochemistry. Cell migration, colony formation, wound-healing, and Transwell matrix penetration assays, revealed that upregulation of CEP55 promoted PANC cells proliferation, migration, and invasion *in vitro*, whereas knockdown of CEP55 attenuated it. In an *in vivo* murine model, CEP55 overexpression accelerated PANC cells tumourigenicity, together with upregulation of the protein levels of invasion-related proteins matrix metalloproteinase (MMP)2, MMP9, and proliferation-related protein Cyclin D1. Downregulation of CEP55 had the reverse effect. Moreover, the nuclear factor κB (NF-κB)/IκBα signalling pathway, which was activated in CEP55-transduced PANC cells and inhibited in CEP55-silenced PANC cells, contributed to CEP55-mediated PANC cell aggressiveness. This study provided new insights into the oncogenic roles of CEP55 and the mechanism by which the NF-κB pathway is hyperactivated in patients with PANC, indicating that CEP55 is a valuable prognostic factor and a potential therapeutic target in PANC.

## Introduction

Pancreatic cancer (PANC) is one of the most aggressive human malignancies, with a median survival of 3–6 months and an overall 5-year survival rate of less than 5%^1, 2^. The poor prognosis is mainly attributed to the high aggressiveness of PANC and the absence of symptoms in the early stage, resulting in locally advanced or metastatic disease at diagnosis^3, 4^. Conventional chemotherapy remains the major option for most PANC patients and surgical resection provides the only chance to cure patients with PANC; However, it provides limited overall survival benefit^5, 6^. Therefore, there is an urgent need to develop new diagnostic biomarkers and novel therapeutic strategies to improve the clinical outcome of PANC^7^.

Nuclear factor κB (NF-κB) has been identified as a key player in the regulation of caner development^8^. Recent research has shown that constitutive activation of the NF-κB pathway is tightly associated with tumourigenesis, migration, and invasion in human carcinomas, including PANC^9, 10^. Up to 70% of pancreatic ductal adenocarcinoma shows a constitutive activation of the NF-κB pathway, which contributes to epithelial-mesenchymal transition, migration, and invasion^9, 11, 12^. NF-κB Pathway activation induced by insulin-like growth factor-binding protein (IGFBP2) drives epithelial-mesenchymal transition and invasion in pancreatic ductal adenocarcinoma^13^. Stimulation of the NF-κB pathway mediated by p-21 activated kinase 4 (PAK4) promotes proliferation and survival of pancreatic cancer cells^14^. Thus, blocking this pathway might prove clinically effective in inhibiting tumour development and offer valuable therapeutic targets for PANC.

Centrosomal protein 55 (*CEP55*), one of the centrosomal family proteins, is identified as a microtubule-bundling protein that participants in critical cell functions, such as cell growth, transformation, and cytokinesis^15, 16^. Increasing evidence shows that CEP55 is overexpressed in premalignant lesions of the colon and in several solid tumours, including human colon cancer, hepatocellular carcinoma, and bladder cancer^17–19^. Importantly, overexpression of CEP55 promoted cell cycle transition in human gastric cancer^15^; whereas knockdown of CEP55 inhibited cell growth in breast cancer^20^ and gastric cancer^15^, decreased cellular motility and invasion in ovarian cancer cells^21^, and induced cell apoptosis in glioma^22^. These results suggested that CEP55 might serve an oncogenic role and could be a potential target for tumour treatment. However, the clinical significance and biological role of CEP55 in PANC remain largely unclear.

In the present study, we provided evidence that CEP55 overexpression increased the activity of NF-κB and promotes PANC cell proliferation, migration/invasion, and tumourigenicity *in vitro* and *in vivo*, which conferred poor prognosis in patients with PANC. In contrast, blocking the NF-κB pathway suppressed the CEP55-induced aggressiveness of PANC cells. The results provided new insight into the mechanisms underlying the hyperactivation of NF-κB pathway in PANC and suggest that CEP55 might be a valuable prognostic factor and potential target in PANC therapy.

## Materials and Methods

### Cell culture

Normal pancreas cell line hTERT-HPNE (Human Pancreatic Nestin Expressing cells) and eight different human PANC cell lines (Panc 03.27, Capan-1, Capan-2, SW1990, HPAF-II, Panc 10.05, BxPC-3, and CFPAC-1) were purchased from the American Type Culture Collection (ATCC, Manassas, VA, USA). The cells were grown in Dulbecco’s modified Eagle’s medium supplemented with 10% foetal bovine serum (HyClone, Logan, UT) and 100 units penicillin-streptomycin at 37 °C with 5% CO_2_ atmosphere in a humidified incubator.

### Clinical specimens

One hundred and twenty-six archived paraffin-embedded PANC specimens were obtained from the Union Hospital, Tongji medical college, Huazhong university of science & technology from 2005 to 2010. Another nine PANC tissues and three adjacent non-tumour tissues frozen in liquid nitrogen were collected for further quantitative real-time reverse transcription PCR (qRT-PCR), western blotting, and immunohistochemical assays. No patients had received any anti-tumour treatments before biopsy. For the use of these human materials, prior consent and approval from the Institutional Research Ethics Committee of Huazhong University of Science and Technology were obtained, informed consent was obtained from the patients involved in the research, and all experiments were carried out in accordance with relevant guidelines and regulation. The clinical information for the patient samples is summarized in Table S1.

### RNA extraction, reverse transcription, and qRT-PCR

Total RNA was extracted from freshly frozen samples or cells using the TRIzol reagent (Invitrogen). Total RNA was reverse-transcribed using a First Strand cDNA Synthesis Kit (Invitrogen). The real-time PCR reactions were conducted using Platinum SYBR Green qPCR SuperMix-UDG reagents (Invitrogen) on an Applied Biosystems 7500 Sequence Detection system. All reactions were done in triplicate and reactions without reverse transcriptase were used as negative controls. The human α-tubulin gene was used as the endogenous control and the 2^−ΔΔCT^ equation was used to calculate the relative expression levels. The primers used to detect gene expression are shown in supplemental Table S3.

### Matrix Metalloproteinase Activity Assays

MMP activity (MMP2 and MMP9) was measured by a fluorogenic peptide substrate (R&D Systems), according to the protocol recommended by the manufacturer. Briefly, the MMP substrate was diluted in TCN buffer (150 mmol/L NaCl, 10 mmol/L CaCl_2_, 50 mmol/L Tris-HCl; pH 7.5), and then was added to the supernatants (preactivated by aminophenylmercuric acetate for 1 hour) before incubation at 37 °C. After 30 minutes, total MMP activity was determined on a fluorometer (FLX 800 Microplate Fluorescence Reader; Bio-Tek Instruments, Winooski, VT).

### Western blotting analysis

Total cell lysate was extracted using radioimmunoprecipitation assay (RIPA) lysis buffer (Beyotime Biotechnology). Approximately 30 µg of protein was separated by 10% SDS-PAGE and transferred onto a polyvinylidene fluoride (PVDF) membrane. After blocking with 5% skimmed milk, the membranes were incubated overnight with primary antibodies against CEP55 (Abcam, ab170414), MMP2 (Abcam, ab37150), MMP9 (Abcam, ab38898), Cyclin D1 (Abcam, ab134175), p-IKKβ (Abcam, ab59195), IKKβ (Abcam, ab32135) and IκBα (Abcam, ab32518), followed by incubation with secondary antibodies. Human α-tubulin (Abcam, ab7291) or GAPDH (Abcam, ab8245) were used as the endogenous references, according to the details. Immunoreactive protein bands were visualised using the ECL method (Invitrogen), according to the manufacturer’s recommendations.

### Immunohistochemical assay

Immunohistochemical analysis was performed on formalin-fixed, paraffin-embedded tissues. Antigen retrieval was performed by heating the slides at 95 °C in citrate buffer (pH 6.0) for 15 minutes before staining. The following primary antibodies were used: anti-CEP55 (Abcam, ab170414), anti-Cyclin D1 (Abcam, ab134175), and anti-MMP9 (Abcam, ab38898). Biotinylated Dolichos biflorus agglutinin (DBA) lectin (Vector Labs) was used at 1:250 in HEPES/NaCl. Staining of CEP55 was quantified using H-scores calculated from the staining intensity (0–3+) and percentage of stained cells (0–100%). The images were captured using the AxioVision Rel.4.6 computerized image analysis system (Zeiss).

### Plasmids, retroviral infection, and transfection

The cDNAs of the human *CEP55* or *IκBα* genes were amplified by PCR and cloned into the lentiviral vector pSin-EF2. Two human *CEP55*-targeting short hairpin RNA (shRNA) sequences (RNA#1: CCCAAGTGCAATATACAGTATCTCGAGATACTGTATATTGCAC TTG and RNA#2: GCAGCGGGAAGTCTATGTAAACTCGAGTTTACATAGACTTCCC GCT) were cloned into the pSuper-retro-puro vector to generate pSuper-retro-*CEP55*-RNAi(s). Retroviral production and infection were performed as described previously^23^. Stable cell lines that expressed *CEP55* or *CEP55*-shRNAs were selected for 10 days using 0.5 mg/mL puromycin.

### MTT assay and colony formation assay

PANC cells were seeded at 1500 cells per well in 96-well plates after transfection. The MTT (3-(4,5-Dimethylthiazol-2-Yl)-2,5-Diphenyltetrazolium Bromide) assay was performed to test cell viability at 1, 2, 3, 4, and 5 days, and the absorbance was measured at 490 nm using a spectrophotometric plate reader. For the colony formation assay, cells were plated at 500 cells per well in six-well plates after transfection, and cultured for 14 days. Colonies were fixed with methanol, stained with 0.5% crystal violet, and counted under an inverted microscope.

### Transwell matrix penetration assay

Cells (1 × 10^4^) were plated on the top side of a polycarbonate Transwell filter coated with Matrigel in the upper chamber of the BioCoat™ Invasion Chambers (BD, Bedford, MA, USA) and incubated at 37 °C for 22 h, followed by removal of cells inside the upper chamber with cotton swabs. Invaded cells on the lower membrane surface were fixed in 1% paraformaldehyde, stained with haematoxylin, and counted (10 random fields per well at 100× magnification).

### Anchorage-independent growth assay

Five hundred cells were trypsinised and suspended in 2 ml completed medium plus 0.3% agar (Sigma). The agar-cell mixture was plated on top of a bottom layer with 1% agar completed medium mixture. After 10 days, viable colonies larger than 0.1 mm were counted. The experiment was carried out independently three times for each cell line.

### Tumour xenografts

Capan-1 cells stably expressing *CEP55* or *shCEP55* (3 × 10^6^ cells/mouse) were injected subcutaneously into 4-week-old nude mice (Center for Experimental Animal of Guangzhou University of Chinese Medicine, Guangzhou, China). Stable *CEP55*-expressing Capan-1 cells and *IκBα-*lentivirus were co-injected subcutaneously into another group of nude mice. Tumour nodules were examined every three days, and were evaluated using the following formula: tumour volume = (Width^2^ × Length)/2. Mice were sacrificed 30 days after inoculation, and then the tumours were excised, weighed, and fixed for immunohistochemical staining. Animal protocols received the approval of the Institutional Animal Care and Use Committee of Huazhong University of Science and Technology. All experiments were carried out in accordance with relevant guidelines and regulation.

### Luciferase assay

Three thousand cells were seeded in 48-well plates and allowed to settle for 24 h. One hundred nanogrammes of luciferase reporter plasmids or the control luciferase plasmid plus 10 ng pRL-TK renilla plasmid (Promega) were transfected into PANC cells using the Lipofectamine 3000 reagent (Invitrogen). Luciferase and renilla signals were determined 24 h after transfection using a Dual Luciferase Reporter Assay Kit (Promega). Three independent experiments were performed and the data are presented as means ± standard deviation (SD).

### Electrophoretic mobility shift assay

The electrophoretic mobility shift assay (EMSA) was performed using the LightShift Chemiluminescent EMSA Kit from Thermo Scientific, according to the manufacturer’s standard protocol. EMSA DNA probes were: NF-κB: sense, 5′-AGTTGAGGGGACTTTCCCAGGC-3′, antisense, 5′-GCCTGGGAAAGTCCCCTCAAC-3′; OCT-1: sense, 5′-TGTCGAATGCAAATCACTAGAA-3′, antisense, 5′-TTCTAGTGATTTGCATTCGACA-3′.

### Microarray data processing and visualization

Microarray data were downloaded from The Cancer Genome Atlas (TCGA) database (http://cancergenome.nih.gov/). Analysis of *CEP55* expression in PANC tissues compared with that in normal pancreas tissues was determined using a published microarray-based high-throughput assessment (n = 191, *P* < 0.001; NCBI/GEO/GSE71729). The data were downloaded from NCBI and analysed using SPSS 19.0 software. Gene Set Enrichment Analysis (GSEA) was performed using GSEA 2.0.9 (http://www.broadinstitute.org/gsea/).

### Statistical analysis

Continuous variables are expressed as mean values ± SD, except where the SEM was used. The statistical tests for data analysis included Student’s two-tailed *t*-test, Chi-squared (χ^2^) tests, Fisher’s exact test, log-rank test, and Spearman correlation analysis. Univariate and multivariate statistical analysis were performed using a Cox regression model. Statistical analyses were performed using the SPSS 19.0 statistical software package; *P* < 0.05 was considered statistically significant.

## Results

### Elevated CEP55 expression in PANC correlated with poor prognosis

**Figure 1:**
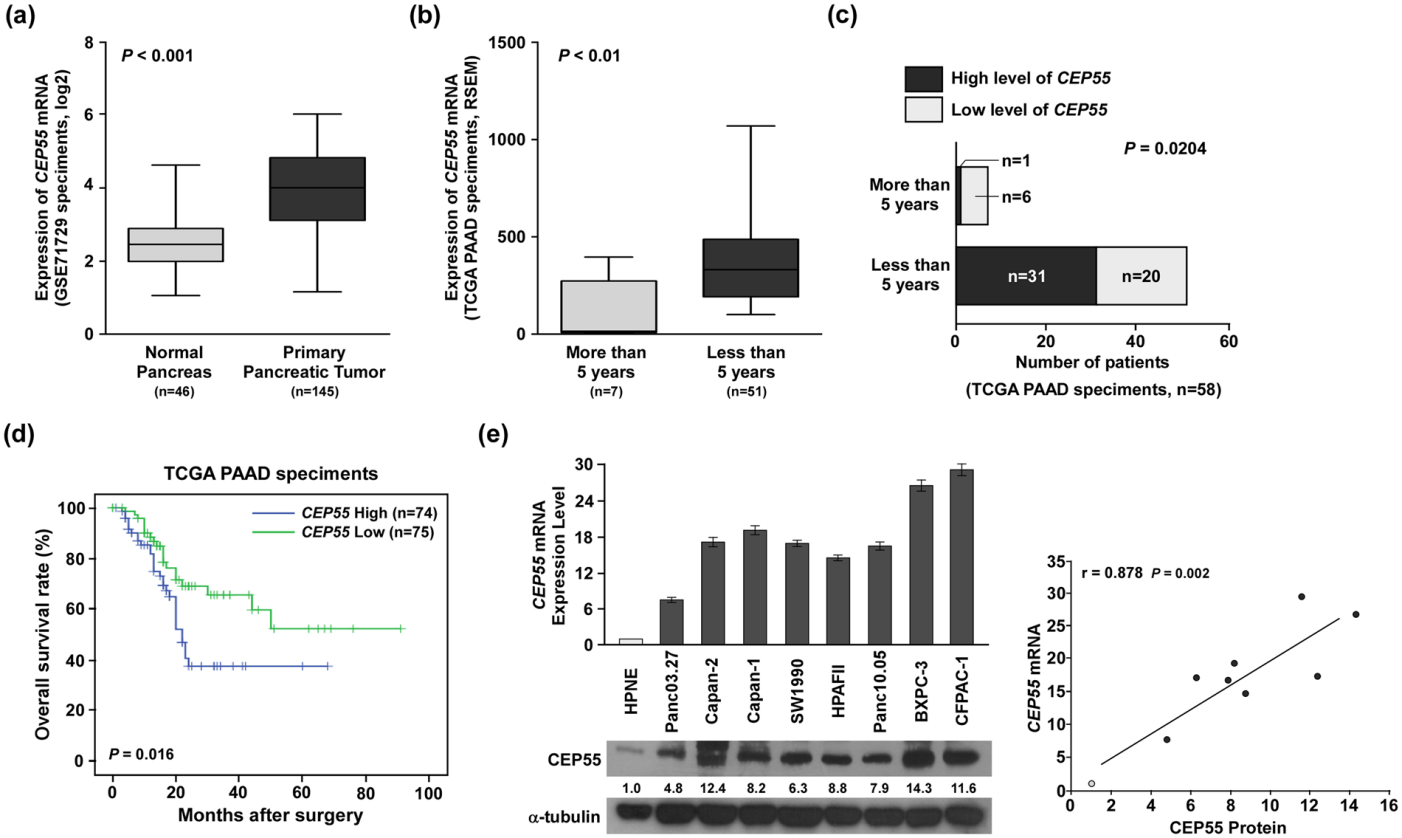
Expression levels of *CEP55* mRNA correlated inversely with overall survival in patients with pancreatic cancer. (**a**) Quantification analyses of *CEP55* mRNA expression between 46 normal pancreas tissues and 145 primary pancreatic tumour specimens from the Oncomine database (GSE71729 specimens). (**b**) Quantification analyses of *CEP55* mRNA expression between seven pancreatic cancer (PANC) specimens with more than 5-year survival and 51 PANC specimens with less than 5-year survival from the TCGA database. (**c**) Statistical correlation between the *CEP55* level and 5-year survival rates in patients with PANC. (**d**) Kaplan-Meier analysis showing associations of overall survival with relatively high and low expressions of *CEP55* mRNA. (**e**) Quantitative real-time reverse transcription-PCR (qRT-PCR) and western blotting detection of *CEP55* mRNA and protein in normal human pancreatic nestin expressing cells (HPNE) and cultured pancreatic cancer cell lines (Panc 03.27, Capan-1, Capan-2, SW1990, HPAF-II, Panc 10.05, BxPC-3, and CFPAC-1) (left panel). The Spearman correlation coefficient was calculated to assess the significance of the association between *CEP55* mRNA and its protein expression levels (right panel). *P < 0.05, **P < 0.01, ***P < 0.001.

To explore the expression of the *CEP55* gene and its clinical significance in human PANC, first, we analysed *CEP55* mRNA expression in primary pancreatic tumours from publicly available PANC datasets (TCGA and GSE71729). The GSEA plot indicated a significant correlation between the *CEP55* mRNA expression level and those genes that were upregulated in PANC gene signatures (*P* < 0.001, Fig. S1a, GRUETZMANN_PANCREATIC_CANCER_UP; TCGA, n = 178, GSE71729, n = 145), which suggested an important role of CEP55 in PANC. Further analysis of the Gene Expression Omnibus (GEO) datasets showed that *CEP55* mRNA was significantly elevated in patients with PANC compared with that in normal individuals (*P* < 0.001, Fig. 1a). In another publicly available PANC dataset (TCGA), *CEP55* mRNA expression was higher in PANC patients with less than 5-year survival than in those with more than 5-year survival time (*P* < 0.01, Fig. 1b). Higher expression of *CEP55* mRNA showed an inverse correlation with the 5-year survival of PANC patients (*P* < 0.05, Fig. 1c). Importantly, Kaplan-Meier analysis revealed that the expression levels of *CEP55* mRNA in PANC specimens were inversely correlated with overall survival for patients with PANC (*P* < 0.05, Fig. 1d), which suggested that the *CEP55* gene might serve as an indicator of poor survival in patients with PANC.

**Figure 2:**
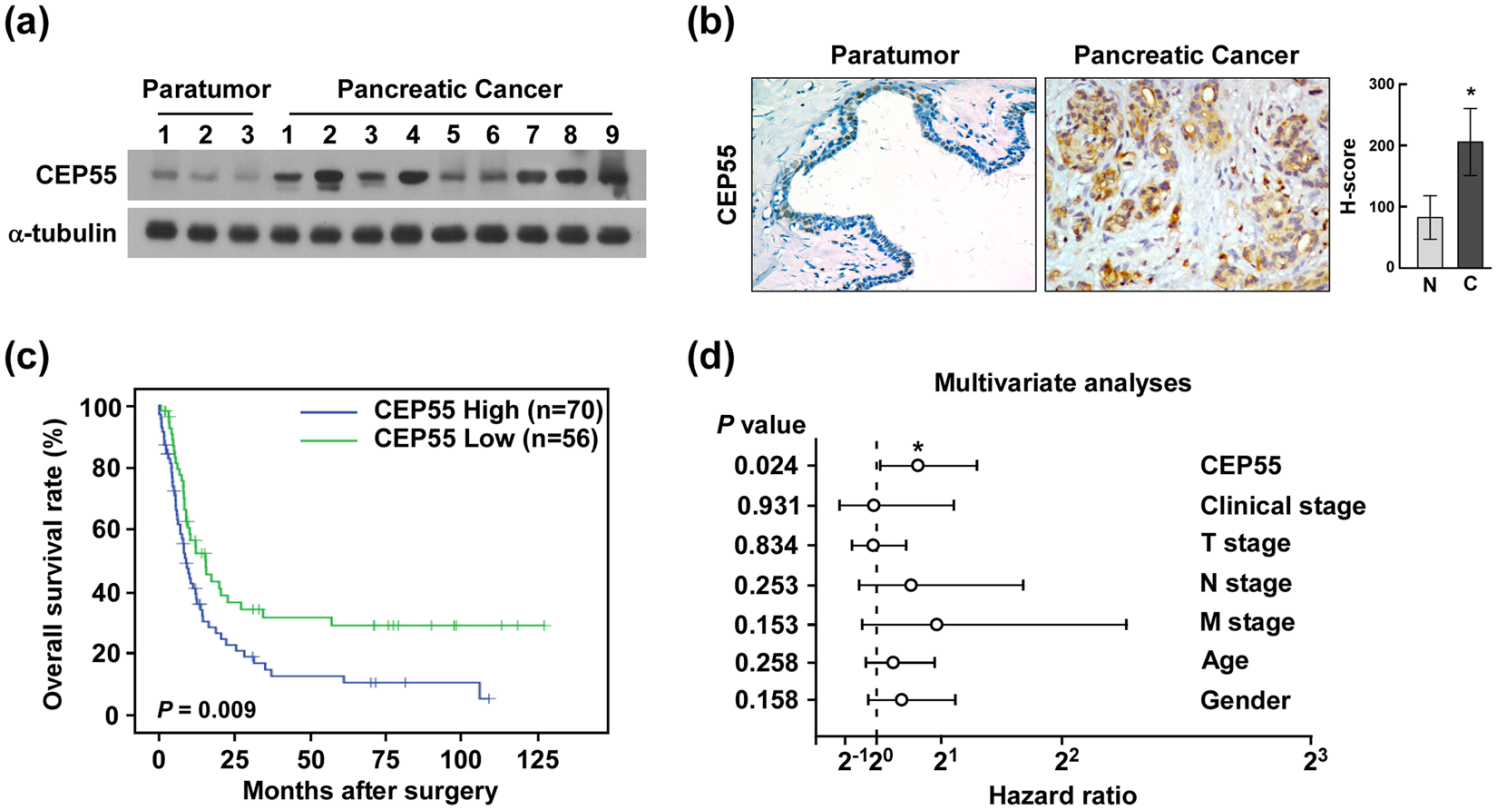
Overexpression of CEP55 protein correlated with poor prognosis in patients with pancreatic cancer (PANC). (**a**) Western blotting detection of CEP55 protein in three adjacent normal pancreas tissues and nine pancreatic cancer tissues. (**b**) Immunochemical analysis of CEP55 protein expression in 126 pancreatic cancer specimens (left panel), magnification ×400. Statistical quantification of the H-score of CEP55 staining of 126 pancreatic cancer specimens (right panel). (**c**) Kaplan-Meier survival curves for 126 pancreatic cancer patients with high CEP55-expressing versus low CEP55-expressing tumours. (**d**) Multivariate analyses between CEP55 protein expression and clinicopathological characteristics of 126 pancreatic cancer specimens. *P < 0.05, **P < 0.01.

To validate the above analyses, we detected *CEP55* mRNA and protein levels in normal pancreas cells (HPNE), PANC cell lines (Panc 03.27, Capan-1, Capan-2, SW1990, HPAF-II, Panc 10.05, BxPC-3, and CFPAC-1), and clinical specimens. Consistent with the published databases, Oncomine and TCGA, the levels of the *CEP55* mRNA and CEP55 protein were significantly higher in PANC cell lines than in HPNE (Fig. 1e, left panel). Correlation analysis also revealed that *CEP55* mRNA and protein levels correlated positively in the nine cell lines (Fig. 1e, right panel). In the clinical specimens, *CEP55* mRNA and protein levels were elevated significantly in the nine PANC tissue samples compared with those in three adjacent non-tumour tissues (Fig. 2a and Fig. S1b).

To further evaluate relationship between CEP55 protein expression and the clinicopathological features of PANC, 126 archived paraffin-embedded PANC specimens were analysed by immunohistochemical staining using an antibody against human CEP55 (Fig. 2b, left panel). Quantitative immunohistochemical analysis revealed that the H-score of CEP55 staining was significantly increased in PANC specimens compared with that in adjacent non-tumour tissues (*P* < 0.05, Fig. 2b, right panel). Among these PANC specimens, 56 cases (44.4%) were identified as relatively low CEP55 expression, while 70 cases (55.6%) showed relatively high CEP55 expression (Table S1). Furthermore, up-regulated CEP55 protein was associated with TNM stage (*P* < 0.05) and clinical stage (*P* < 0.01), but no correlations were found with age or gender (Table S2). Importantly, Kaplan-Meier analysis revealed that the CEP55 protein expression levels in PANC specimens were inversely correlated with survival time (Fig. 2c). Finally, multivariate analysis was performed, which identified CEP55 expression as an independent prognostic factor of patient outcome (Fig. 2d). Taken together, our results indicated that CEP55 might represent a novel and potentially valuable independent biomarker for the prognosis of patients with PANC.

### CEP55 overexpression promoted PANC cells aggressiveness

**Figure 3:**
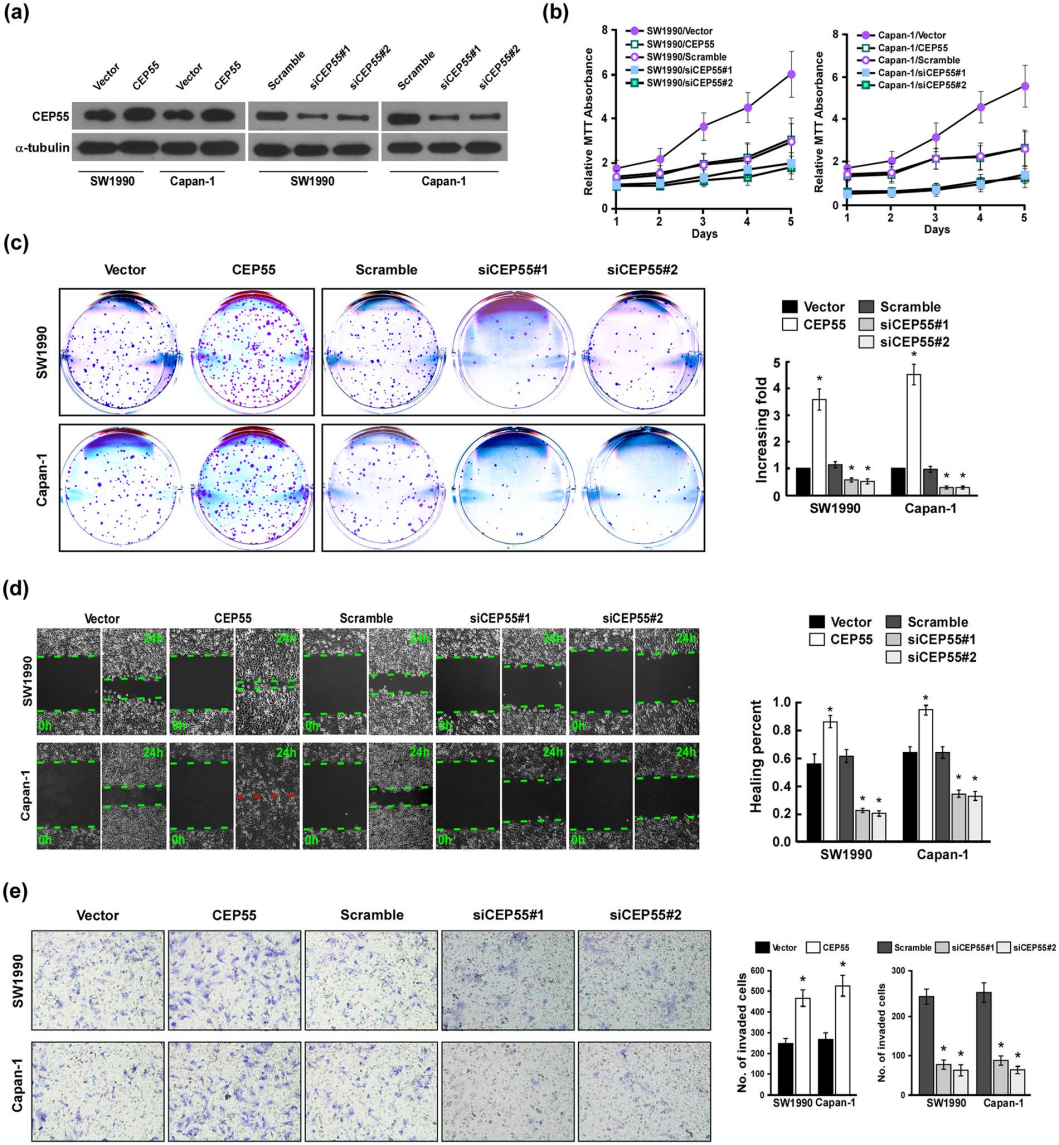
CEP55 overexpression promoted pancreatic cancer (PANC) cells aggressiveness *in vitro*. (**a**) Expression of CEP55 protein as detected by Western blot analysis in SW1990 and Capan-1 cells upon transfection with CEP55 or CPE55 short interfering RNAs (siRNAs). GAPDH was used as the loading control. (**b**) MTT (3-(4,5-Dimethylthiazol-2-Yl)-2,5-Diphenyltetrazolium Bromide) assay of SW1990 and Capan-1 cell growth curves following CEP55 or CEP55 siRNAs transfection. (**c**) Representative micrographs (left panel) and quantification (right panel) of crystal violet-stained SW1990 and Capan-1 cell colonies after 14 day of colony formation. (**d**) Wound-healing assay (left panel) and quantification (right panel) of the wound distance to assess SW1990 and Capan-1 cell migration after transfection with CEP55 or CEP55 siRNAs. (**e**) Representative micrographs (left panel) and quantification of SW1990 and Capan-1 cell invasion in a Transwell matrix penetration assay. The quantification of the invaded cells is represented by the mean of three independent experiments. Bars represent the mean ± SD of three independent experiments. *P < 0.05.

PANC is highly aggressive; therefore, we next investigated the effects of CEP55 on the proliferation, migration, and invasion of PANC cells. Initially, SW1990 and Capan-1 cell lines expressing CEP55 moderately were selected as cell models in this study according to the CEP55 expression in the PANC cell lines (Fig. 1e). CEP55 expression was detected by qRT-PCR and Western blot in SW1990 and Capan-1 cell lines upon transfected with CEP55 or CEP55 siRNA (Fig. 3a, Fig. S1c). Subsequently, MTT and colony formation assays were performed to evaluate the roles of CEP55 in PANC cell proliferation. The MTT assay revealed that CEP55 upregulation promoted SW1990 and Capan-1 cell proliferation (Fig. 3b). In the colony formation assay, CEP55 overexpression significantly increased the viability of SW1990 and Capan-1 cells, which formed more and larger colonies (*P* < 0.05, Fig. 3c). Conversely, silencing endogenous CEP55 in these cells markedly suppressed their proliferation and viability (Fig. 3b,c).

Next, wound-healing and Transwell matrix penetration assays were carried out to assess the effect of CEP55 on migration and invasion. As shown in Fig. 3d, CEP55 overexpression markedly accelerated SW1990 and Capan-1 cell migration compared with the vector control-transduced cells in the wound-healing assay, whereas silencing endogenous CEP55 in these cells reduced their migration capability (*P* < 0.05, Fig. 3d). In the Transwell matrix penetration assay, CEP55-overexpressing SW1990 and Capan-1 cells showed significantly increased invasion abilities, while CEP55 knockdown decreased it (*P* < 0.05, Fig. 3e). Take together, CEP55 overexpression promoted the proliferation, migration, and invasion of PANC cells *in vitro*, whereas downregulation of CEP55 expression attenuated it.

### CEP55 upregulation accelerated PANC cells tumourigenicity

**Figure 4:**
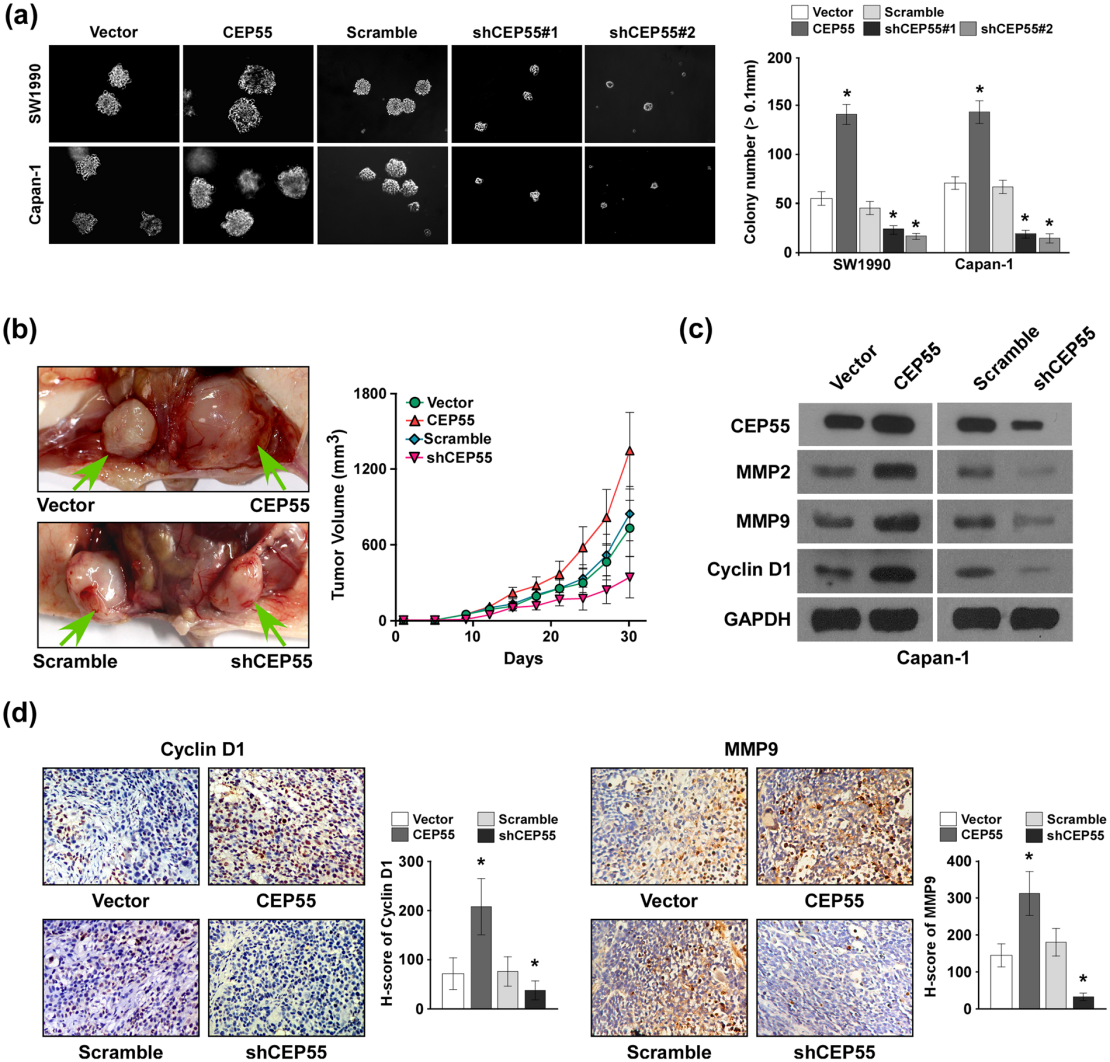
CEP55 overexpression accelerated pancreatic cancer (PANC) cells tumourigenicity *in vivo*. (**a**) Representative micrographs (left panel) and quantification (right panel) of colonies >0.1 mm formed in the anchorage-independent growth assay. (**b**) Subcutaneous tumour xenograft model in nude mice; representative images of the tumours formed by CEP55- or shCEP55-transduced Capan-1 cells (left panel, n = 5 per group). Growth curves for tumour formation after implantation of Capan-1 cells transfected with CEP55 or shCEP55 (right panel). (**c**) Western blotting detection of MMP2, MMP9, Cyclin D1 and CEP55 in tumour tissues derived from these mice. Glyceraldehyde-3-phosphate dehydrogenase (GAPDH) was used as a loading control. (**d**) Representative images of an immunohistochemical assay of Cyclin D1 (left panel) and MMP9 (right panel) expression in tumour tissues derived from these mice, magnification ×400. Statistical quantification of the H-score of Cyclin D1 and MMP9 staining in the indicated tissue samples. Bars represent the mean ± SD of three independent experiments. *P < 0.05.

To further investigate the biological role of CEP55 in PANC cell aggressiveness, we evaluated the effect of CEP55 on the tumourigenicity in PANC cells. As shown in Fig. 4a, CEP55 overexpression markedly increased the anchorage-independent growth ability of SW1990 and Capan-1 cells in soft agar, as indicated by the increased colony number and size, whereas silencing endogenous CEP55 decreased tumourigenicity in these cells, which showed that CEP55 promoted PANC cells tumourigenicity *in vitro*.

The tumourigenicity capability of CEP55 was further examined using an *in vivo* murine model. SW1990/vector, SW1990/CEP55, Capan-1/scramble, or Capan-1/shCEP55 cells were injected subcutaneously into nude mice (n = 5 per group), and the growth morphologies of the implanted tumours were examined (Fig. 4b, left panel). The tumour growth rates were faster and their volumes were larger in CEP55-implanted mice than in the vector-implanted mice (Fig. 4b, right panel). Conversely, CEP55-silenced cells presented much slower tumour growth rates and formed smaller tumours (Fig. 4b). The tumour tissues were derived from these mice for further western blotting and immunohistochemical assays. Western blotting (Fig. 4c) and immunohistochemical assays (Fig. 4d) revealed the upregulation of invasion-related proteins MMP2 and MMP9, and proliferation-related protein Cyclin D1 in the CEP55-overexpressing tumour tissues, which coincided with the aggressive phenotype displayed by Capan-1/CEP55 cells. In contrast, MMP2, MMP9, and Cyclin D1 levels were decreased in tumour tissues with downregulated CEP55 expression (Fig. 4c,d). These results indicated that CEP55 accelerated PANC cells tumourigenicity and contributed to tumour progression *in vivo*.

### The NF-κB pathway contributed to CEP55-mediated PANC aggressiveness and tumourigenicity

**Figure 5:**
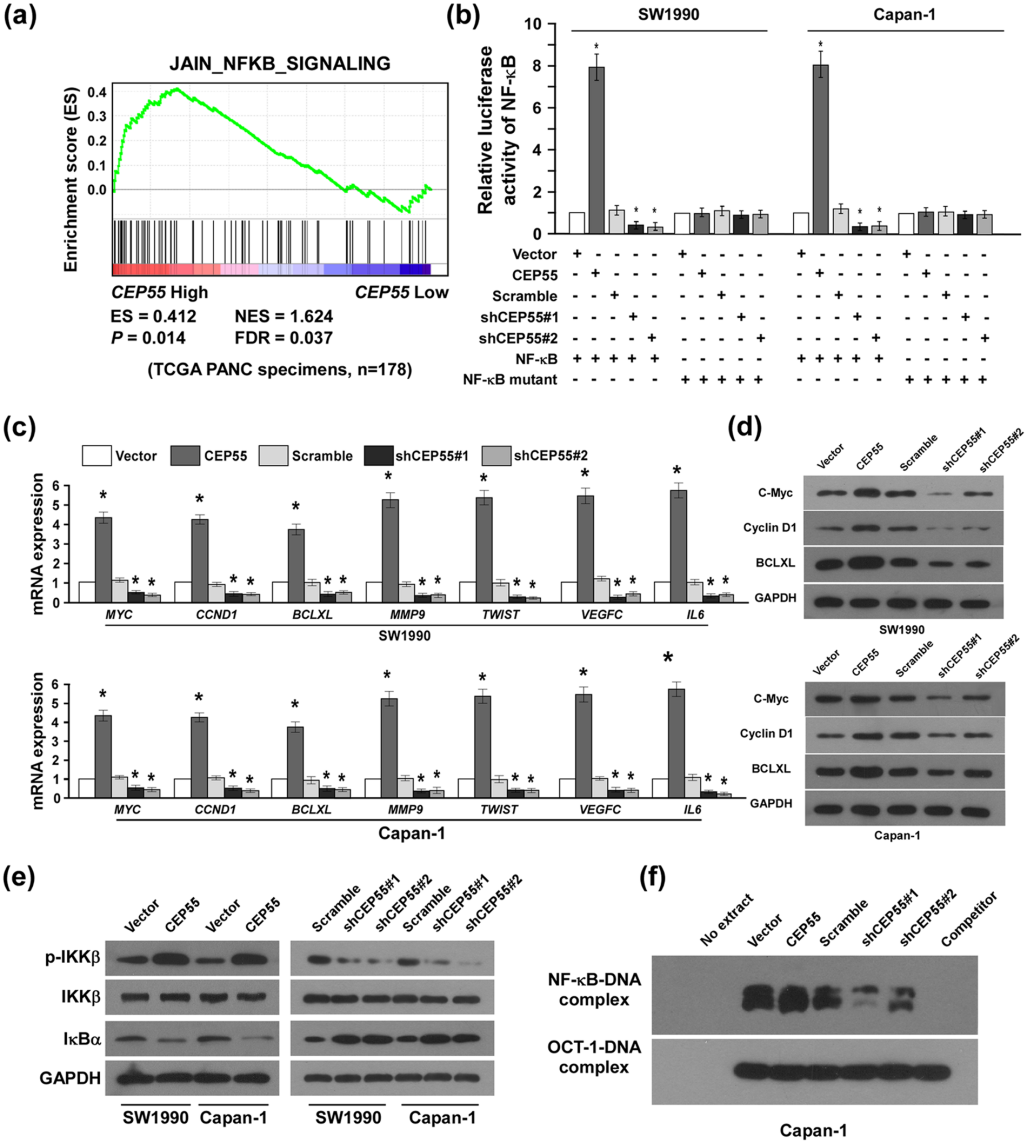
CEP55 activated NF-κB signalling. (**a**) Gene set enrichment analysis (GSEA) plot indicating a significant correlation between the *CEP55* gene expression level and nuclear factor kappaB (NF-κB)-activated gene signatures (JAIN_NF-κB_SIGNALING). (**b**) Luciferase-reported NF-κB activity in SW1990 and Capan-1 cells transduced with CEP55 or shCEP55. (**c**) Changes in mRNA expression of NF-κB downstream targets (*MYC*, *CCND1*, *BCLXL*, *MMP9*, *TWIST*, *VEGFC* and *IL6*) in the indicated cells assessed by real-time PCR. (**d**) Changes in protein expression of NF-κB downstream targets (C-Myc, Cyclin D1 and BCLXL) in the indicated cells assessed by western blot. (**e**) Western blotting analysis of p-IKKβ, IKKβ, and IκBα expression in SW1990 and Capan-1 cells transduced with CEP55 or shCEP55. (**f**) The endogenous NF-κB activity in the indicated cells detected by an electrophoretic mobility shift assay (EMSA). The Oct-1-DNA complex served as a control.

To identify the major pathways contributing to CEP55-mediated aggressiveness of PANC cells, we carried out correlation analysis between *CEP55* expression and the possible signalling pathways in the GSEA database. *CEP55* mRNA expression correlated positively with NF-κB-activated gene signatures (Fig. 5a), suggesting that *CEP55* might activate the NF-κB signalling pathway. Indeed, ectopic expression of *CEP55* increased significantly the NF-κB-driven luciferase reporter activity, whereas CEP55-knockdown decreased it (Fig. 5b). However, neither overexpression nor knockdown of CEP55 had any effect on the luciferase activities driven by mutated NF-κB (Fig. 5b), which further demonstrated that CEP55 plays an important role in modulation of NF-κB pathway. Furthermore, the expression of seven classically recognized NF-κB downstream targets, including *MYC*, *CCND1*, *BCLXL*, *MMP9*, *TWIST*, *VEGFC*, and *IL6*, were elevated on mRNA level in CEP55-overexpressing PANC cells (Fig. 5c). In contrast, CEP55 downregulation had the opposite effect (Fig. 5c). Further protein expression levels of C-Myc, Cyclin D1 and BCLXL were examined by western blotting assay. Consistently, we found that overexpression of CEP55 dramatically increased but silencing CEP55 decreased the expression of these NF-κB targets (Fig. 5d). These results suggested an important role of CEP55 in the activation of the NF-κB pathway.

IKKβ serves as a central intermediate signalling molecule in the activation of the NF-κB pathway; we assessed the effect of CEP55 modulation on IKKβ transcriptional activity in PANC cells. As expected, the expression levels of phosphorylated-IKKβ increased markedly in CEP55-transduced PANC cells, but decreased in CEP55-silenced cells (Fig. 5e). Consistently, levels of IκBα, the inhibitory protein of NF-κB, were reduced in CEP55-overexpressing PANC cells, whereas they were increased in CEP55-knockdown cells (Fig. 5e). Furthermore, EMSA revealed that NF-κB DNA-binding activity was increased drastically in CEP55-transduced cells and decreased in CEP55-silenced cells (Fig. 5f), suggesting that NF-κB signalling plays a critical role in promoting CEP55-mediated PANC aggressiveness.

**Figure 6:**
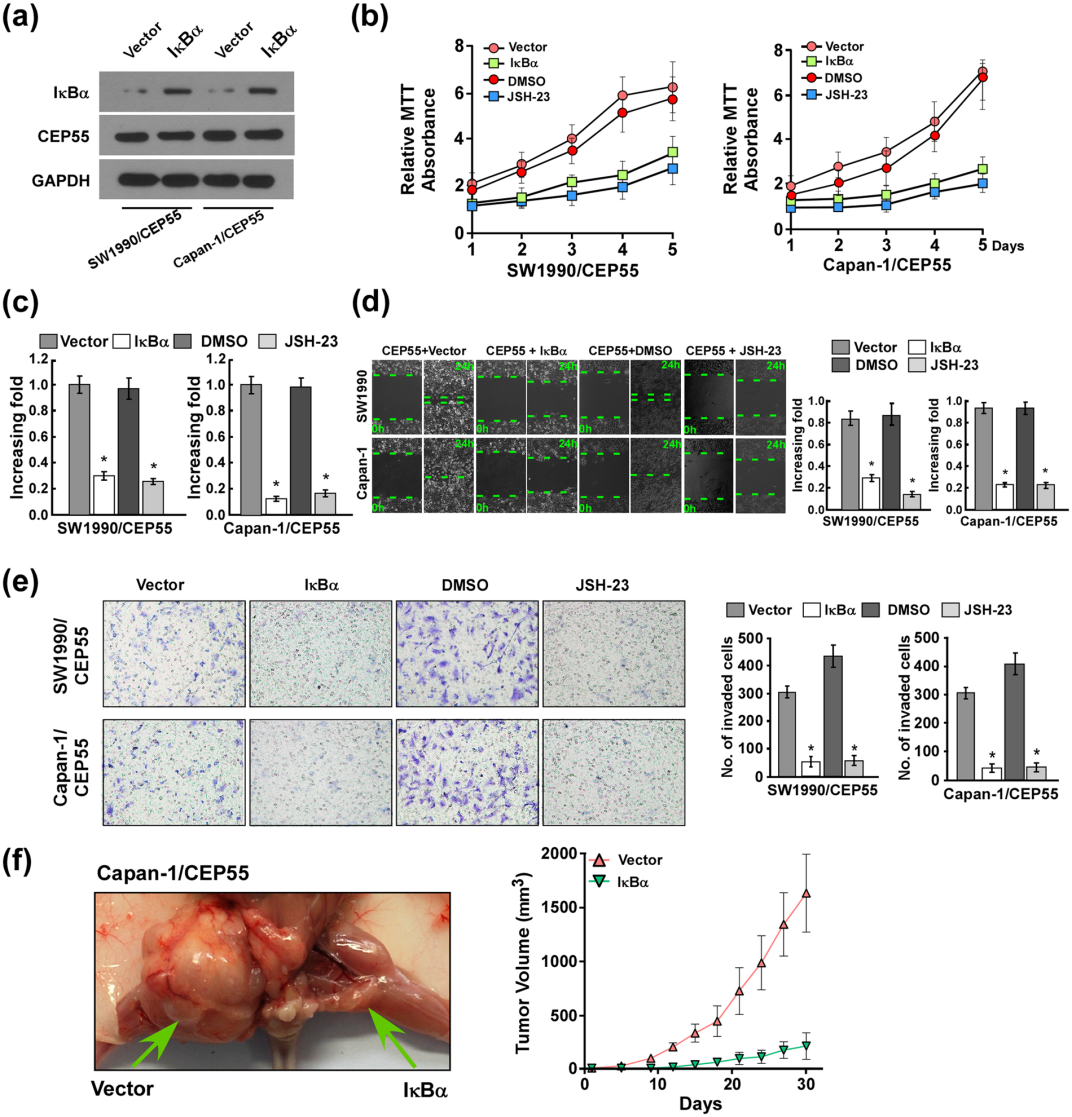
The nuclear factor kappaB (NF-κB) pathway contributed to CEP55-mediated aggressiveness and tumourigenicity of pancreatic cancer (PANC) cells *in vitro* and *in vivo*. (**a**) The protein expression of IκBα transfected into CEP55-overexpressing PANC cells is detected by western blotting assay. (**b**) MTT assay of CEP55-overexpressing SW1990 and Capan-1 cells transduced with IκBα or JSH-23. (**c**) Quantification of crystal violet-stained colonies of CEP55-overexpressing SW1990 and Capan-1 cells transduced with IκBα or JSH-23 after 14 days of colony formation. (**d**) Wound-healing assay assessment of CEP55-overexpressing SW1990 and Capan-1 cell migration after transfection with IκBα or JSH-23. (**e**) Quantification of CEP55-overexpressing SW1990 and Capan-1 cell invasion in a Transwell matrix penetration assay. The quantification of invaded cells represents the mean of three independent experiments. (**f**) Subcutaneous tumour xenograft model in nude mice; representative images of the tumours formed by CEP55-overexpressing Capan-1 cells transfected with IκBα (left panel, n = 5). Growth curves for tumour formation after implantation of CEP55-overexpressing Capan-1 cells transfected with IκBα (right panel). Bars represent the mean ± SD of three independent experiments. *P < 0.05.

To further validate that CEP55-mediated PANC cell aggressiveness acts via NF-κB activation, we blocked the NF-κB pathway by reintroducing the IκBα cDNA retrovirally or introducing the exogenetic NF-κB inhibitor JSH-23 into CEP55-overexpressing PANC cells. As expected, both IκBα and JSH-23 inhibited the stimulatory effect of CEP55 overexpression on NF-κB activation. MTT and colony formation assays showed that IκBα and JSH-23 suppressed the enhanced capabilities of proliferation and viability in PANC cells that were induced by CEP55 overexpression via NF-κB activation (Fig. 6a–c), whereas NF-κB pathway activation could partially rescue the reduced proliferation upon depletion of CEP55 (Fig. S2). Moreover, IκBα and JSH-23 also inhibited the elevated migratory and invasive capability in CEP55-overexpressing cells, as determined by wound-healing and Transwell matrix penetration assays (Fig. 6d,e). Importantly, the enhanced tumourigenicity was reversed by IκBα and the tumour volume was much smaller compared with that in CEP55-overexpressing Capan-1 cells (Fig. 6f). These results revealed that NF-κB pathway contributed to CEP55-mediated PANC aggressiveness and tumourigenicity *in vitro* and *in vivo*.

**Figure 7:**
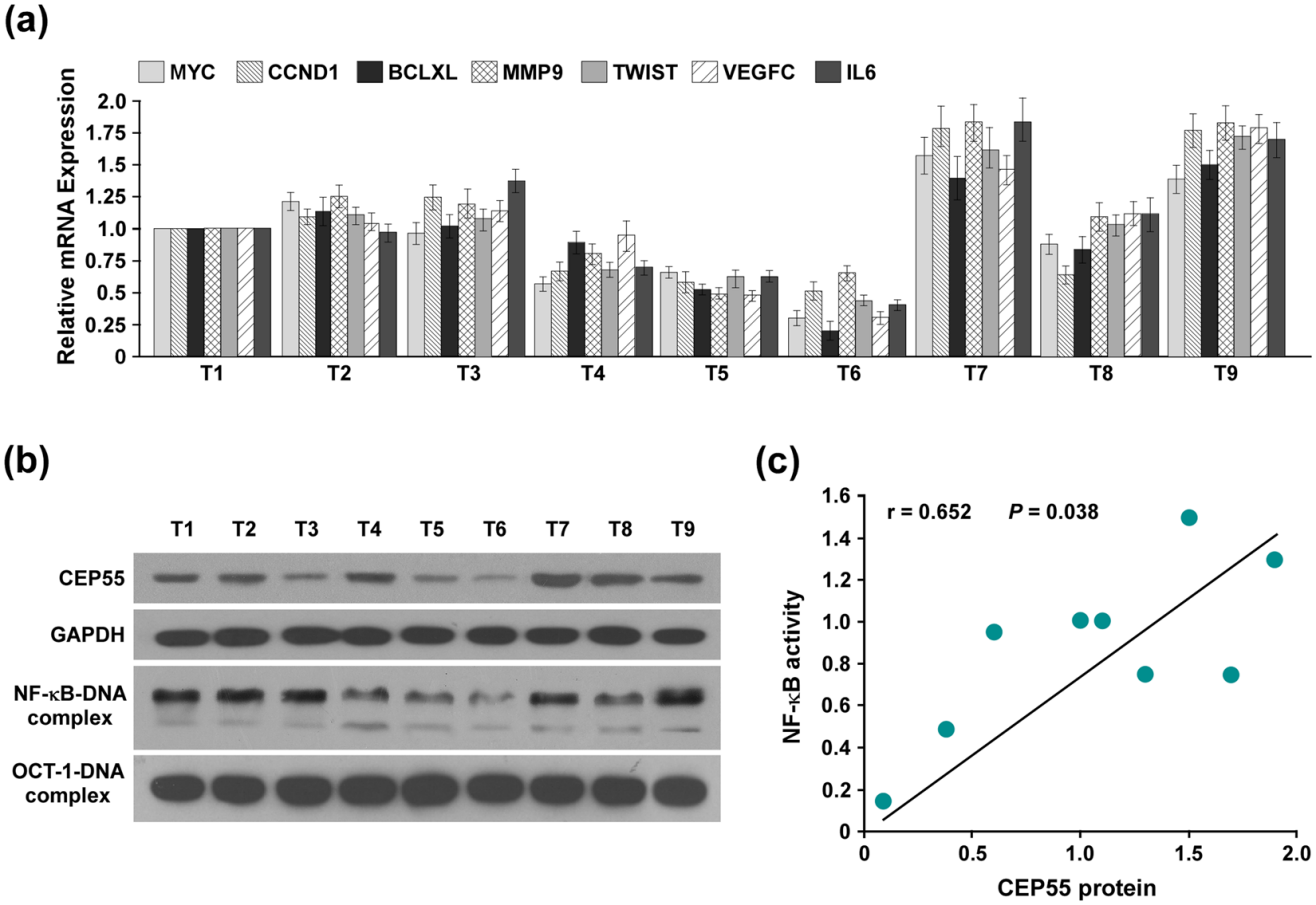
CEP55 expression was clinically correlated with the activation of nuclear factor kappaB (NF-κB) signalling in patients with pancreatic cancer (PANC). (**a**) Changes in mRNA expression of NF-κB-regulated genes (*MYC*, *CCND1*, *BCLXL*, *MMP9*, *TWIST*, *VEGFC* and *IL6*) in nine pancreatic cancer tissues assessed by real-time PCR. (**b**) Western blotting analysis of CEP55 expression in these nine pancreatic cancer tissues (Upper panel). The endogenous NF-κB activity in these nine pancreatic cancer tissues detected by an electrophoretic mobility shift assay (EMSA) (Lower panel). (**c**) The Spearman correlation coefficient was calculated to assess the significance of association between CEP55 protein and NF-κB activity. *P < 0.05.

Finally, we examined whether CEP55 and NF-κB activation identified in PANC cells were clinically relevant. As shown in Fig. 7a and b, the PANC patients with relatively high CEP55 protein levels tended to have higher NF-κB DNA-binding activities and expressions of NF-κB target genes, including *MYC*, *CCND1*, *BCLXL*, *MMP9*, *TWIST*, *VEGFC and IL6* (Fig. 7a,b and Fig. S3). Further correlation analysis revealed that CEP55 protein expression correlated positively with NF-κB activity (Fig. 7c), which further supported the notion that CEP55 could activate NF-κB pathway clinically. Taken together, these results suggested that CEP55 promoted PANC cells aggressiveness and tumourigenicity, probably by activating the NF-κB signalling pathway in patients with PANC.

## Discussion

The key finding of the present report is that CEP55 promotes PANC cell proliferation, migration/invasion, and tumourigenicity by activating the NF-κB signalling pathway. Elevated CEP55 expression in patients with PANC correlated with poor prognosis, whereas blocking the NF-κB pathway inhibited the CEP55-mediated aggressiveness of PANC cells, indicating that CEP55 represents a valuable prognostic indicator of outcome or is a potential therapeutic target for patients with PANC.

CEP55 is a key regulator of cytokinesis and promotes cell cycle transition^15^. It is located in the mitotic spindle during prometaphase and metaphase, and is recruited into the midbody during cytokinesis^16, 24^. Its aberrant expression has been proposed as an early event in tumourigenesis^25^. Increasing evidence shows that CEP55 has an oncogenic role and its overexpression correlates markedly with tumour stage, aggressiveness, and poor prognosis across multiple tumour types, such as gastric carcinoma, breast cancer, and ovarian carcinoma^15, 20, 21, 26^. In this study, CEP55 was detected as overexpressed in PANC cell lines and in patients with PANC. CEP55 promoted the proliferation, migration, and invasion in PANC cells *in vitro*. In an *in vivo* murine model, CEP55 overexpression accelerated PANC cells tumourigenicity, which was associated with upregulated levels of invasion-related proteins MMP2 and MMP9, and the proliferation-related protein Cyclin D1. CEP55 downregulation had the opposite effect. MMP2 and MMP9, as regulators of cell adhesion and invasion, are involved in augmenting the invasive capability of tumour cells, and correlate with the degree of histological malignancy and clinical outcome^27, 28^, while Cyclin D1 is associated with the proliferative ability of tumours. We believe that CEP55 accelerates PANC cell tumourigenicity *in vivo* by enhancing their proliferative and invasive capabilities, and functions as a novel oncogene in PANC aggressiveness.

NF-κB plays crucial roles in the regulation of inflammatory and immune responses, as well as in biological processes central to development of malignancies^29^. In about 70% pancreatic ductal adenocarcinoma, the NF-κB pathway is activated constitutively and is associated tightly with tumourigenesis, migration, and invasion^9, 11, 12^. By contrast, NF-κB signalling is key to immune function and is likely necessary for antitumor immunity^30, 31^. This presents a dilemma when designing therapeutic apprcoaches that target NF-κB. However, there is growing interest in identifying novel modulators to inhibit NF-κB activity, because impeding different steps of the NF-κB pathway has the potential to slow tumour growth, progression, and resistance to chemotherapy. In this study, the NF-κB pathway was hyperactivated in patients with PANC and contributed to CEP55-mediated proliferation, migration/invasion, and tumourigenicity in PANC cells *in vitro* and *in vivo*. Downregulated expression of CEP55 reduced, whereas CEP55 overexpression increased, the phosphorylation levels of IKKβ, an IκBα kinase, leading to IκBα degradation and release of NF-κB from the cytoplasm to the nucleus, where NF-κB activated the transcription of its downstream target, such as *MYC*, *CCND1*, *BCLXL*, *MMP9*, *TWIST*, *VEGFC*, and *IL6*. In patients with PANC, CEP55 protein expression and NF-κB activation were shown to be clinically relevant. Importantly, blocking the NF-κB pathway in PANC cells reversed CEP55-mediated PANC aggressiveness and tumourigenicity substantially. Taken together, these results revealed a novel molecular mechanism by which the NF-κB/IκBα pathway is hyperactivated in PANC and suggested that CEP55 or NF-κB/IκBα are potential targets for pharmacological intervention in PANC.

CEP55 was identified as a direct transcriptional target of forkhead box M1 (FOXM1)^32^. FOXM1 is overexpressed in PANC^33^ and its downregulation inhibits PANC cell growth, migration, and invasion, partly through the downregulation of NF-κB^33^. Another study revealed that CEP55 promotes FOXM1 expression^34^. Furthermore, both CEP55 and FOXM1 are regulated negatively by p53, a potent tumour suppressor^35, 36^, while mutations of p53 are frequently found in pancreatic ductal adenocarcinoma^37^. One hypothesis states that p53 inactivation or mutation leads to constitutive activation of a feedback loop between CEP55 and FOXM1, resulting in the enhanced aggressiveness in PANC via activation of the NF-κB pathway. However, further in-depth studies are needed to determine the precise molecular regulation of CEP55 and NF-κB, and their crosstalk, in cell growth, migration/invasion, and tumourigenicity in human PANC.

In summary, we demonstrated that CEP55 upregulation promotes PANC cell aggressiveness, such as cell proliferation, migration/invasion, and tumourigenicity. The aggressiveness-promoting role of CEP55 in PANC is associated with the degradation of IκBα and activation of NF-κB transactivity. Understanding the biological function of CEP55 in PANC will not only advance our knowledge of the mechanisms underlying CEP55 aggressiveness, but also will establish CEP55 and NF-κB/IκBα as significant prognostic factors or potential therapeutic targets to treat PANC.

## Electronic supplementary material

Supplemental information
