# Supplementary material for: Centrosomal protein 55 activates NF-κB signalling and promotes pancreatic cancer cells aggressiveness
Source: Sci Rep. 2017 Jul 19;7:5925. doi: 10.1038/s41598-017-06132-z (PMC5517556; doi:10.1038/s41598-017-06132-z)
Supplement: Supplementary file 1 — Supplemental information [file 41598_2017_6132_MOESM1_ESM.pdf]

Centrosomal protein 55 activates NF-κB signalling and promotes pancreatic cancer cells aggressiveness

Tao Peng[1#], Wei Zhou[1#], Feng Guo[1], He-shui Wu[1], Chun-you Wang[1], Li Wang[2*] & Zhi-yong Yang[1*]

[1]Department of Pancreatic Surgery, Union Hospital, Tongji Medical College, Huazhong University of Science and Technology, 1277 Jiefang Avenue, Wuhan 430022, China.

[2]Department of Emergency Surgery, Union Hospital, Tongji Medical College, Huazhong University of Science and Technology, 1277 Jiefang Avenue, Wuhan 430022, China.

[#]These authors contributed qually to this work.

[*]Correspondence and requests for materials should be addressed to Z.Y. (email: zhiyong_yang134@163.com) and L.W. (email: huanhuan1229@163.com)

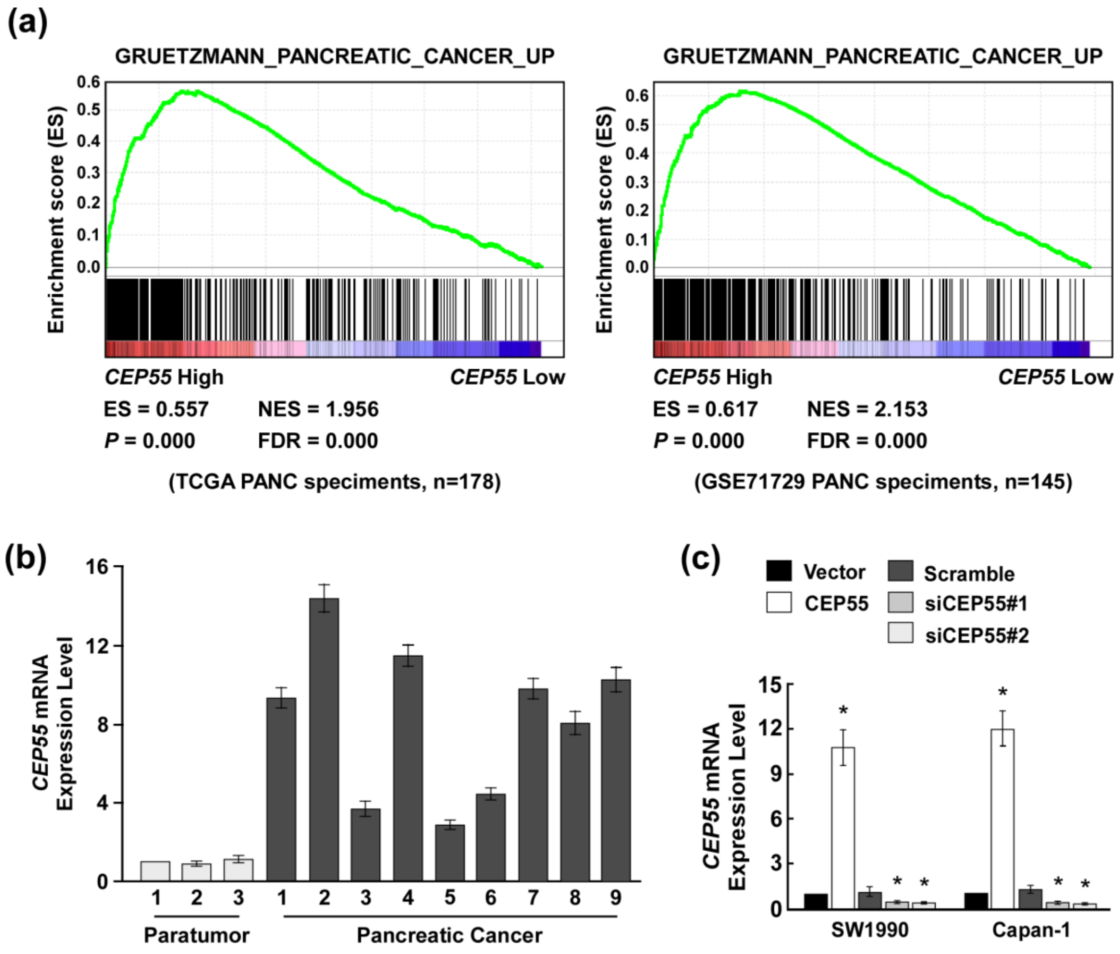

Figure S1. Elevated *CEP55* mRNA expression in pancreatic cancer.

(a) Gene set enrichment analysis (GSEA) plot indicating a significant correlation between *CEP55* mRNA expression level and those genes up-regulated in pancreatic cancer gene signatures (GRUETZMANN_PANCREATIC_CANCER_UP; TCGA, n=178, GSE71729, n=145). (b) Quantitative real-time reverse transcription-PCR detection of *CEP55* mRNA in three normal pancreas tissues and nine pancreatic cancer tissues. (c) Expression of *CEP55* mRNA as detected by qRT-PCR analysis in SW1990 and Capan-1 cells upon transfection with CEP55 or CPE55 siRNAs. GAPDH was used as the loading control. Bars represent the mean ± SD of three independent experiments. *$P < 0.05$.

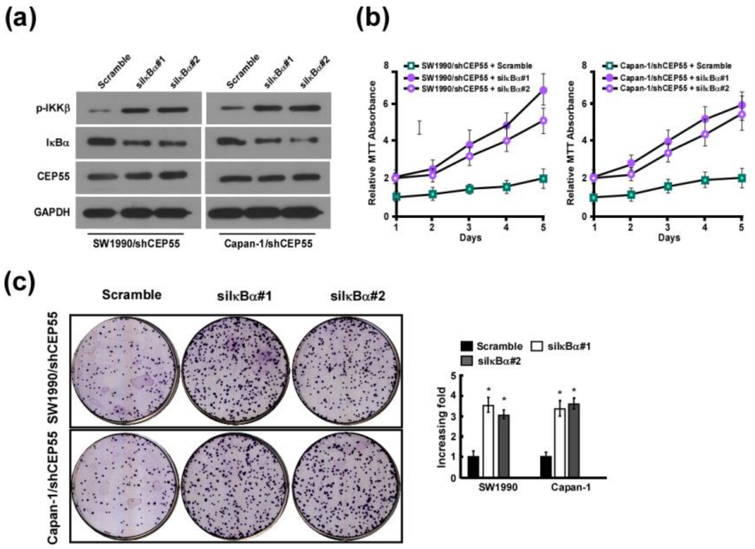

Figure S2. NF-κB activation could partially rescue the reduced proliferation of PANC cells upon depletion of CEP55.

(a) Western blotting analysis of p-IKKβ and IκBα expression in CEP55-downregulating SW1990 and Capan-1 cells upon transfection with siIκBα. (b) MTT assay of CEP55-downregulating SW1990 and Capan-1 cells transduced with siIκBα. (c) Representative micrographs (left panel) and quantification (right panel) of crystal violet-stained colonies of CEP55-knockdown SW1990 and Capan-1 cells transduced with siIκBα after 14 days of colony formation.

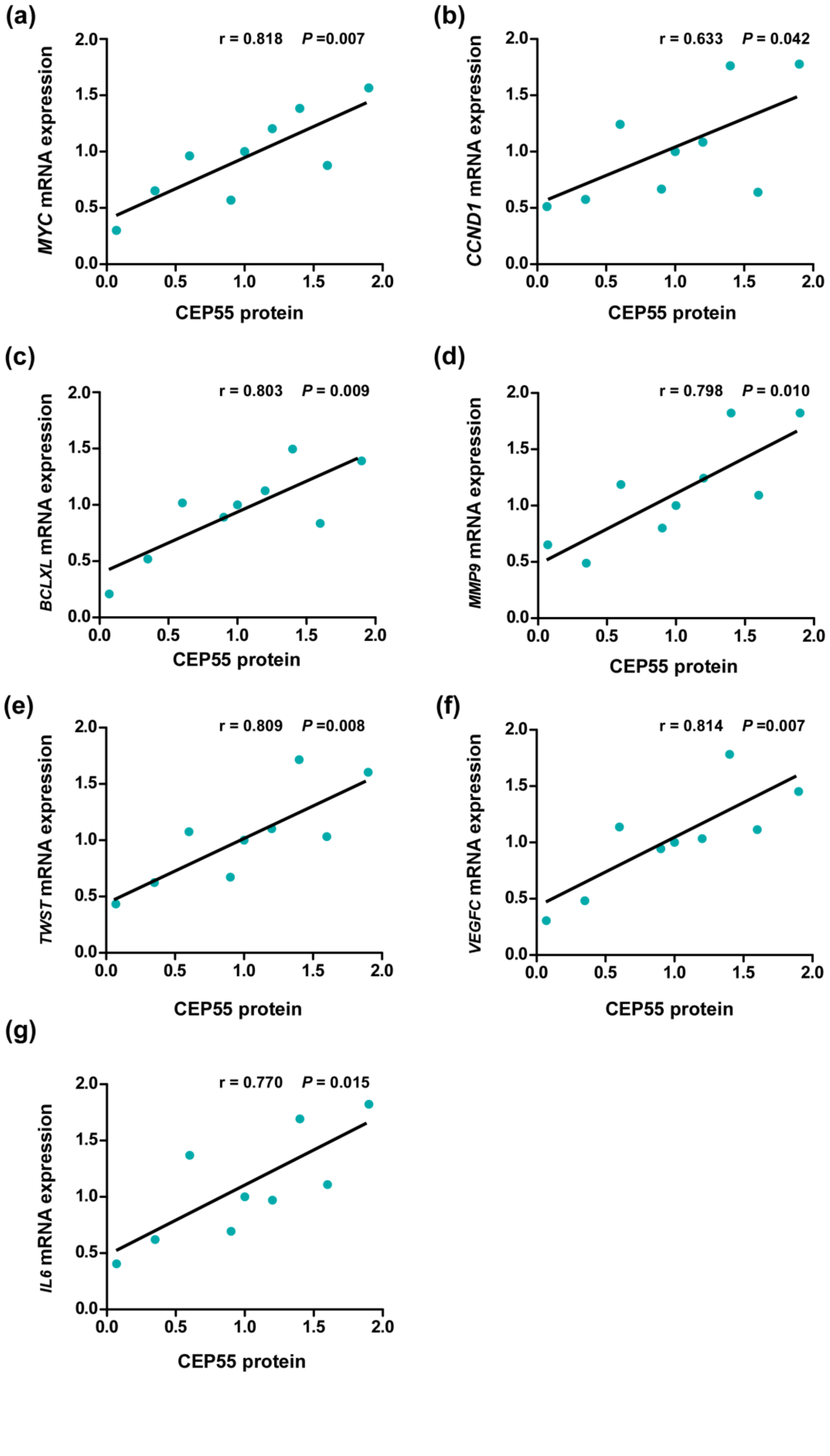
(a)
r = 0.818 P =0.007
MYC mRNA expression
CEP55 protein
(b)
r = 0.633 P = 0.042
CCND1 mRNA expression
CEP55 protein
(c)
r = 0.803 P = 0.009
BCLXL mRNA expression
CEP55 protein
(d)
r = 0.798 P = 0.010
MMP9 mRNA expression
CEP55 protein
(e)
r = 0.809 P =0.008
TWST mRNA expression
CEP55 protein
(f)
r = 0.814 P =0.007
VEGFC mRNA expression
CEP55 protein
(g)
r = 0.770 P = 0.015
IL6 mRNA expression
CEP55 protein
0.0
0.5
1.0
1.5
2.0

Figure S3. The Spearman correlation coefficient was calculated to assess the significance of association between CEP55 protein and NF-κB downstream targets in 9 PANC specimens. ****P*<0.05.**

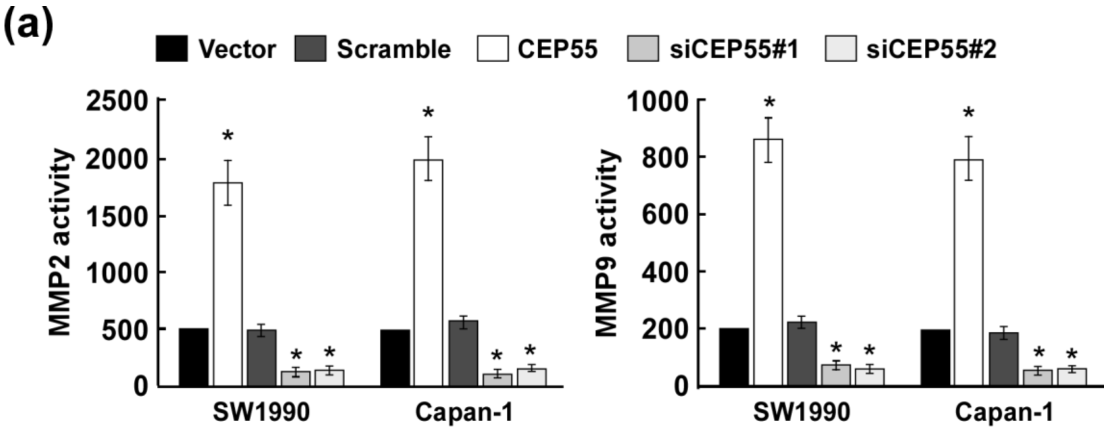

Figure S4. The activity of MMP2 and MMP9 in pancreatic cancer cells upon transfection wieh CEP55 or siCEP55.

Table S1. Clinicopathological characteristics of PANC patient samples.

Characteristics	Number of cases
**Gender**	
Male	80
Female	46
**Age(years)**	
>57	59
≤57	67
**Clinical Stage**	
I	47
II	34
III	34
IV	11
**T classification**	
T1	25
T2	47
T3	34
T4	20
**N classification**	
N0	81
N1	45
**M classification**	
M0	115
M1	11
**Expression of CEP55**	
Relatively high expression	70
Relatively low expression	56
**Vital status**	
Survive	35
Mortality	91

Table S2. Correlation between CEP55 Expression and Clinicopathologic Charateristics of Pancreatic Cancer Patient.

**Characteristic**	**CEP55 Protein level**		**Chi-square test**
	**High**	**Low**	***P* value**
**Age (> 57 versus ≤ 57 years)**	32/38	27/29	*0.858*
**Gender (M versus F)**	48/22	32/24	*0.198*
**Clinical stage (I/II versus III/IV)**	38/32	43/13	*0.009*
**T classification (T1/T2 versus T3/T4)**	34/36	38/18	*0.032*
**N classification (N0 versus N1)**	39/31	42/14	*0.027*
**M classification (M0 versus M1)**	60/10	55/1	*0.022*

Table S3. Primers used for detecting genes expression.

Gene	Real-time PCR primer
CEP55	5’- TTGGAACAAC AGATGCAGGC-3’ and<br>5’- GAGTGCAGCAGTGGGACTTT -3’
MYC	5’-TCAAGAGGCGAACACACAAC-3’ and<br>5’-GGCCTTTTCATTGTTTTCCA-3’
IL6	5’-TCTCCACAAGCGCCTTCG-3’ and<br>5’-CTCAGGGCTGAGATGCCG-3’
BCLXL	5’-TCCTTGTCTACGCTTTCCACG-3’ and<br>5’-GGTCGCATTGTGGCCTTT-3’
VEGFC	5’-GTGTCCAGTGTAGATGAACTC-3’ and<br>5’-ATCTGTAGACGGACACACATG-3’
MMP9	5’-TTGGTCCACCTGGTTCAACT-3’ and<br>5’-ACGACGTCTTCCAGTACCGA-3’
TWIST	5’-GGAGTCCGCAGTCTTACGAG-3’ and<br>5’-TCTGGAGGACCTGGTAGAGG-3’
CCND1	5’-AACTACCTGGACCGCTTCCT-3’ and<br>5’-CCACTTGAGCTTGTTCACCA-3’
GAPDH	5’-ATTCCACCCATGGCAAATTC-3’ and<br>5’-TGGGATTTCCATTGATGACAAG-3’
